# Dermatology

**Published:** 1878-07

**Authors:** 


### Dermatology.

Treatment of Pityriasis Capitis and of Alopecia.—Malassez (*Journal de Médecine*, Dec., 1877).

The facts placed beyond doubt by Malassez in regard to this affection, are, first the constant existence and very great abundance of a certain species of fungus, and second, the absence of this fungus, or at least, its great rarity where there is no pityriasis, or where it no longer exists, as well as in other squamous affections, such as eczema, psoriasis, and ichthyosis. This fungus consists solely of spores. They are found in the corneous layer of the epidermis, where they form horizontal layers or veritable heaps between the diverse layers of this horny portion. At the same time a vesicular alteration of the epidermic cells, already described by Ranvier, is proven.

M. Malassez concludes from these facts, that pityriasis results from the invasion of the hairy scalp by this fungus. 1st. Arriving upon a soil favorable to their development (arthritic subjects) the spores multiply, infilrate themselves into the corneous layer of the epidermis, and there separate into layers. 2d. This invasion produces in the tissues an irritation, a reaction which manifests itself by the vesicular state of the epidermic cells, a new cause of desquamation.

From this double cause, then, external and internal, and from the double mechanism, direct and indirect action, result important practical conclusions as regards treatment. This should be local and general. The local treatment alone will occupy us at present; it should be essentially antiparasitic, and it is necessary in applying it, not to lose sight of the nature and seat of the parasite. Being a fungus, the parasite may be destroyed by such agents as turpeth mineral, sublimate, etc. This parasite, being situated in the corneous layers of the epidermic layers which are all impregnated with fatty matters, it is necessary to make the parasiticide penetrate them. The following treatment carried out carefully succeeds perfectly.

1st. Every second day combing with a fine comb, and soaping the head with common soap. This removes mechanically the scurf, and removes the fatty matters from the hair, and opens up the retreats of the fungus to the action of the parasiticide. With long-haired persons this is not practicable. Men and children should have their hair cut closely. As we cannot usually ask women and young girls to make a sacrifice of their long hair, frictions with commercial alcohol should take the place of the soaping.

2d. When the hair is well dried, friction should be made to the scalp with an ointment composed of equal parts of cocoa butter, castor oil, and oil of sweet almonds, containing one part of turpeth mineral to fifteen of the excipient. Only small quantities should be made at a time, as it soon becomes rancid. In place of this, benzoated lard may be employed as the excipient, though the author prefers the vegetable fats mentioned. Great care should be used in applying the ointment so as to reach every spot of the scalp, and it should be well rubbed in. Large quantities should be used, and frictions made every day. After a week or two of treatment, combing and cleansing of the scalp may be made less frequently, say twice a week, and later but once a week. The ointment may also be applied but two or three times a week, though it is well to continue its use for some time. Amelioration comes soon, complete cure less so, and requires considerable persistence in treatment.

For pityriasis of the beard the ointment would be inconvenient, and may be replaced by alcoholic solutions of corrosive sublimate one part to 500 or 1,000, according to circumstances. A small brush (a soft tooth brush is useful) is saturated with this solution, and rubbed into a small portion of the affected skin at a time, then wet again and applied to a new spot. After all is done, wait a few moments, and then wipe off any excess of liquid on the beard.

The combs and brushes used, should be frequently cleaned with potash, or they will become new sources of contagion.

The alopecia. which succeeds pityriasis, results from the formation of an epidermic plug in the upper portion of the hair follicle, this being an obstacle to the normal exit of the hair. Irritation follows in the deeper portion of the follicle, then hypertrophy of the walls, and finally, obliteration of the hair follicle. After a time, only a fibrous cord is left in its place. Treatment at that stage is of course useless. But during the developmental stage one may hope to arrest the disease, and even to make the hairs more vigorous :

1st. By unplugging the orifices of the hair follicles; 2d. By curing the pityriasis, which affects the superficial regions; 3d. By counteracting the irritation in the deeper parts. For the last condition we may add to the turpeth mineral ointment, from two to four parts of tincture of cantharidis to thirty of the ointment. As the required effect is produced slowly, the cantharidis should be continued long after the pityriasis has disappeared. A weaker ointment should then be used without the turpeth mineral, or but very little of it, say half a part to one part of the mineral and thirty of the excipient, the cantharidis remaining the same. Gallic acid ointment is also useful in these cases. The patient should be informed that friction will at first cause an increased fall of the hair, but it is only temporary, and the diseased and loosened hairs will be replaced by more vigorous ones.

PITYRIASIS PILARIS.—*Journal de Médecine*, Dec., 1878.

M. Richard, in a thesis just published, describes this rare and interesting affection. The principal elements of this very remarkable work were drawn from the service of M. Besnier, whose interne Richard was, and he proves that this affection attacks the entire epidermic system, following it into its ramifications to the bottom of the sebaceous glands and hair follicles, affecting the nails, the hairs, and perhaps even the sudoriparous glands.

Pityriasis pilaris, whatever may be its mode of origin, is characterized by the presence of large red patches covered with scales, patches often large enough to cover the entire trunk without the slightest interval of sound skin. If the patient be examined closely then, there will be seen on other parts of the body small eminences traversed by a hair, and in the palms of the hands, and in certain cases, even the soles of the feet, there are seen large scales; the nails are hypertrophied, and the face is covered with a sebaceous coating. All these lesions are symmetrical. These large red surfaces are a little rough to the touch, but become smooth when freed of their scales, which is easily done. This surface presents neither crusts nor vesicles, nor the slightest moisture. The trunk is most often the seat of this desquamation, and it never descends lower than the thighs. Besides, upon the hairy regions, there is observed another lesion which characterizes pityriasis pilaris; it is the epidermic cones. These pathological productions occupy the regions covered with hairs, except the scalp, the beard, and the pubis. They are especially distinct on backs of the fingers and extend from them to the forearm. These epidermic eminences exist exclusively around the hairs. They consist of two portions, the one intradermic, the part toward the base of the follicle; the other, extradermic, with truncated summit, which looks toward the free extremity of the hair. They are sometimes traversed by a hair, surrounding it like a ring on the finger. Sometimes, on the contrary, they cover the hair, imprisoning it and preventing its escape. If then the little eminence be picked off, the hair set at liberty unrolls itself and straightens. These little eminences give to the skin the appearance of "goose-flesh," and to the hand the sensation of a rasp. These cones thus constituted, do not occupy the regions covered by the pityriasic disquamation. They occupy the hairy regions of the hand. They stop at the sides, and in the palm is often seen a lesion of a different kind; it consists in the presence of large epidermic scales which give a horny appearance to the region. The nails themselves are sometimes hypertrophied. At the same time, the hairs are generally diseased and fall in part. To these local phenomena there are few general phenomena to add. These consist solely in certain troubles of sensibility, and sometimes itching. The affection is essentially a chronic one.

These are very briefly the principal characters of pityriasis pilaris which permit it to be distinguished from ichthyosis pilaris-which is nearly always congenital, from psoriasis, and from pity riasis rubra which differs from it, especially in the absence of the epidermic cones. Unfortunately treatment seems to be of little use. M. Richard advises topical applications of glycerole of starch, to which he adds ten per cent. of cherry laurel water to mask the odor of the glycerine and to diminish itching. At the same time alkaline and starch baths may be tried, and Vichy water, Bazin's alkaline syrup, or the arsenate of sodium.

QUINIA-EXANTHEMA.—Kœbner (*Vierteljahresschrift f. Dermatolog. und Syphil.* 1877, No. 4).

In May of last year, we gave the notes of a case of a scarlatina-like eruption following the administration of quinine. Dr. K. observed two similar cases of idiosyncrasy. The symptoms are so exactly like those of the case reported last May, that we need not repeat the full history. Suffice it to say that in the first case reported by K., the exanthema first broke out in June, 1876, after 0.225 grammes of quinine had been taken; it lasted a whole week, and the desquamation continued six weeks. In September of the same year, another eruption after 0.15 quinine; desquamation beginning on the fifth day. In November, a third attack after 0.1 quinine; desquamation began on the third day and lasted two weeks. It will be noticed that the duration of the exanthema bore a decided relation to the dose of quinine taken.

